# A content analysis on the perceptions of LGBTQ+ (centred) health care on Twitter

**DOI:** 10.1111/hex.13631

**Published:** 2022-10-17

**Authors:** Cornelia Van Diepen, Diego Rosales Valdes

**Affiliations:** ^1^ Erasmus School of Health Policy & Management Erasmus University Rotterdam Rotterdam The Netherlands; ^2^ Centre for Person Centred Care University of Gothenburg Gothenburg Sweden; ^3^ Public Partner

**Keywords:** health care, LGBTQ, patient‐centred care, person‐centred care, Twitter

## Abstract

**Background:**

LGBTQ+ individuals have experienced many barriers to receiving quality health care, but the worldwide implementation of person‐centred care should make a positive change. However, as forthright disclosures are difficult to find using traditional methods, novel approaches should be utilized to uncover opinions and experiences on LGBTQ+ health care. Twitter could be a place where people post on this topic.

**Aim:**

This study aimed to explore tweets mentioning LGBTQ+ (centred) health care.

**Methods:**

The methods consisted of an explorative qualitative content analysis of tweets. The tweets were collected between 26 February and 30 March 2021, resulting in 2524 tweets of which 659 were relevant for content analysis.

**Results:**

The results showed an excess of political tweets involving LGBTQ+ health care. Many tweets included general statements on the need for LGBTQ+ health care. The few tweets on personal experiences in LGBTQ+ health care showed the overwhelming need for quality care that has been made difficult by political developments.

**Conclusion:**

Most tweets were made to inform others of the necessity of quality health care for LGBTQ+ individuals, but the utilization of person‐centred care is hardly noticeable.

**Public Contribution:**

This study was conducted with the involvement of a public partner (second author) who contributed to the design, data analyses and writing of the paper. Moreover, this study involves the analysis of data provided by the public and published on social media.

## 1 BACKGROUND

Lesbian, gay, bisexual, transgender, questioning, and otherwise‐identified (LGBTQ+) individuals have difficulty receiving equal treatment in health care compared to their heterosexual, cisgender peers.^1^ The difficulty often lies with not understanding the LGBTQ+ individual's needs or an outright refusal of treatment because of who the individual is.^2^, ^3^ The LGBTQ+ community encompasses about 4%–7% of the population and due to its association with poorer mental health and taboos, this percentage of individuals could be much higher.^4^ A large part of the problem is the lack of competency from healthcare professionals to understand personal beliefs, specific issues, and the importance of an inclusive environment.^3^ Two reviews concluded that healthcare professionals were aware of their limitations in providing appropriate care to LGBTQ+ individuals and expressed that a person‐centred approach to healthcare would increase the quality of care for these patients.^4^, ^5^

In recent decades, person‐centred care (PCC) has become an increasingly discussed concept in health care. Healthcare professionals, patient organizations and policymakers increasingly aspire to PCC to optimize cost containment and quality of care.^6^ PCC is directed at improving the health and recovery process of patients by forming a partnership between the patient (often with relatives) and the healthcare professionals. A central challenge of PCC is how to incorporate the needs of everyone, including the underrepresented voices. When a PCC approach is applied in care for LGBTQ+ individuals, the healthcare professional can understand LGBTQ+ identity development from the perspective of the individual.^7^ PCC therapy includes a focus on interpersonal and intrapersonal difficulties faced in health care such as the experience of stigma.^8^

PCC is an umbrella term to cover an array of different concepts and terms that are often used interchangeably: patient‐centred care, client‐centred care, and family‐centred care.^9^, ^10^, ^11^ This plurality indicates that researchers, healthcare professionals, and patients may not have the same understanding of PCC.

Tweets can hold unexpected potential for exploring frank opinions on contested issues. Twitter is a supply‐based platform on which people offer information and present their opinions to connect with like‐minded people.^12^, ^13^ LGBTQ+ individuals and their allies use Twitter to discuss health and social needs relevant to the population.^14^ This social network platform is used to find news feeds and content relevant to the LGBTQ+ identity that can be difficult to find in offline settings.^15^ The unfiltered nature of Twitter can be a valuable source of insight into the honest opinions of individuals who tend to feel left out when it comes to health.^16^ Twitter contains vast amounts of freely available, user‐generated microblogs and provides real‐time monitoring of public health topics in an efficient and automated manner. Eysenbach defines this monitoring as ‘Infoveillance’ which is ‘the science of distribution and determinants of information in an electronic medium, specifically the Internet, or in a population, with the ultimate aim to inform public health and public policy’.^17^ ^, p.2^ Infoveillance may have many benefits for the field of LGBTQ+ health care as it could provide new insights from voices that would otherwise go unheard. The analysis of the LGBTQ+ health care contents of the tweets can offer both a better understanding of how it is expressed and an indication of why the tweet was made.

Tweeting can be considered a performative platform to self‐identify.^18^, ^19^ By interacting or simply spreading posts, a performance of self is provided for an imagined audience.^13^ Self‐disclosures of personal emotional messages receive the most positive response from the imagined audience.^20^ A study on the #meToo movement in tweets has shown that individuals tweet about their experiences with as much detail as the microblogs of 280 characters will allow.^21^ An empirical study on the Nigerian Queer community showed expressions of their sexualities, as well as purposive identity formations, manifest in both in‐ and out‐group advocacies.^22^ Based on these studies, presenting experiences of health care from the LGBTQ+ community could be found on Twitter. Moreover, the literature has shown how important the experiences of healthcare professionals with LGBTQ+ individuals are and therefore more perspectives need to be considered in this study to portray the perceptions of LGBTQ+ health care.

To get a better perspective on what people are posting about LGBTQ+ (centred) health care, only the content of the tweet is used. This approach allows for the exploration of this topic within tweets. The purpose of this study is to explore the tweets mentioning LGBTQ+ health care on Twitter and uncover if person‐centredness appears in any of these tweets.

## 2 METHODS

This is an explorative qualitative content analysis study on Twitter.

### 2.1 Data collection

The Twitter search was conducted in the Twitter Application Programming Interface (API) called Mozdeh.^23^ The search terms consisted of LGBTQ+ terminology with mentions of health care (see Supporting Information: Appendix File 1 for the full list). These search terms were compiled through public and patient involvement.  Both English and Spanish language search terms were applied as these are the languages most spoken in the United States.

The API collected the tweets posted between 26 February and 30 March 2021. The start date was chosen because this was right after the passing of the ‘Equality Act’ in the United States and a month of data yielded ample tweets for qualitative analysis. This Act outlaws discrimination against the LGBTQ+ community in housing, credit, jury service, public accommodations and federal funding, which includes (gender‐affirming) health care. The Equality Act induced a pushback from several US states with a legislative ban on gender‐affirming health care for minors. These events increased the number of tweets on our topics as people expressed their opinions and experiences on the new situation.

**Figure 1:**
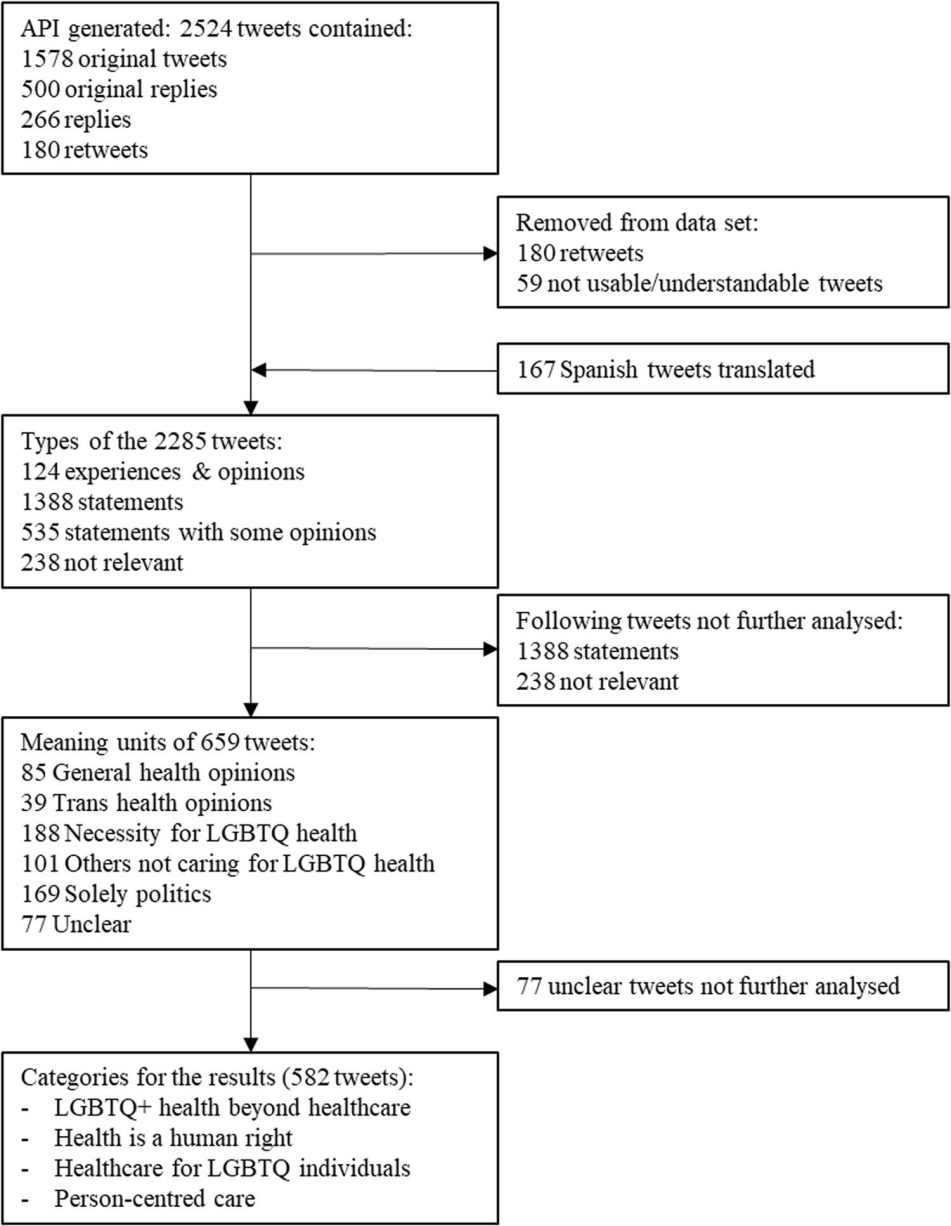
Exclusion diagram

The API generated 2524 tweets consisting of 1578 original tweets (although many copies with the same texts), 500 original comments (these start with @####), 266 replies, and 180 retweets. The retweets were removed from the sample as the content was made by someone else. These remaining tweets tallied a sample of 2344 tweets for the content analysis. This sample contained 167 tweets in the Spanish language which were translated by the second author who is a native speaker. 59 tweets only contained a reference to pages or usernames and were eliminated. The remaining 2285 tweets could be further analysed. The flowchart (Figure 1) shows the steps in the data collection and analysis.

### 2.2 Content analysis

The content analysis was performed following the guidelines described by Graneheim and Lundman.^24^ The tweet's content was read line by line and coded by the first author, whereby meaning units were created and categorized (see flowchart in Figure 1).

The tweets were first selected on their type: opinions and experiences, statements, statements with some opinions or not relevant. The statements and not relevant tweets were removed from further analysis leaving 659 tweets to be classified into the following meaning units: General health opinions (85), Trans health opinions (39), Necessity for LGBTQ health (188), Others not caring for LGBTQ health (101), Solely politics (169), and a rest category where the meaning was unclear (77).

The tweets were then read through again based on their classification by both authors. The tweets need to be presented in a logical order which was achieved through categories going from universal (LGBTQ+ health beyond health care) to specific (person‐centred care). Both authors discussed the analysis, and disagreements were settled through negotiated consensus. The confirmability was improved by using tweets to demonstrate the grounding of the findings in the data. Table 1 presents an example of the coding scheme.

**Table 1:** Example of coding scheme

Tweet	Type	Meaning Unit	Category
Being a minority, part of the lgbtq+ community, and care about mental health sometimes makes me forget that there are still people out there without basic human decency 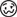 and flaunting it at that crazy	Experiences and opinions	General health	Healthcare for LGBTQ+ individuals
@####### Not as long as the GQP opposes voting equality, racial justice, reproductive rights, LGBTQ rights, a living minimum wage, access to health care for all, fair immigration policies, progressive taxation, etc., and supports insurrection, conspiracy	Statements with some opinions	Solely politics	LGBTQ+ health beyond healthcare
Essentially, there is nothing (exception of a hand full of services) implemented in mainstream primary care mental health for LGBTQ+ community, other than an access target. Sadly the access target is pointless if people are afraid to seek support or speak about their identity.	Statements with some opinions	General health	Healthcare for LGBTQ+ individuals
@##### Let me get this straight, people are upset about a covid vaccine card being against their constitutional rights to enter a privately owned store, but a LGBT child can be denied health care?	Experiences and opinions	Others not caring for LGBTQ health	Health is a human right
@##### Los problemas mentales no tienen género querido. Aquí esto no es de feminismo o machismo. Es alguien que necesitaba atención médica de un psicólogo/psiquiatra y nunca la recibió.	Statements with some opinions	Necessity for LGBTQ health	Health is a human right
@#####Mental health issues do not have gender dear. Here this is not about feminism or male sexism. It is someone who needed medical attention from a psycologist/psychiatrist and never received it			
Great. Now do economic policy, taxes, the environment, welfare, civil rights, voter rights, LGBT, the war on drugs, criminal justice, gun control, abortion, education, health care, defense spending, I'm running out of space, Jesus H. Christ, you guys were wrong on everything	Experiences and opinions	Unclear	‐
March 22‐26 is LGBTQ Awareness Week. Changes in health care, education needed to improve LGBT heart health (website)	Statement	‐	‐

### 2.3 Ethical considerations

Twitter data is supply‐based in that the researchers can only access what others have publicly presented on the platform. When people create a Twitter account, they must agree to the terms and conditions that state that their output can be used by third parties. As Twitter users are often unaware that they agreed to this, certain steps are taken to prevent the Twitter user from harm. There has been much debate and different approaches to the portrayal of tweets in research. Some guidelines dictate that researchers should always ask for consent to quote the tweets and paraphrase all other tweets.^25^ However, there is a selection bias when applying consent^26^ and paraphrasing would contain a level of interpretation that takes away from the word choices of the Twitter users. Therefore, the authors of this study decided to copy the tweets directly from the API to preserve the expression of Twitter users. However, this paper follows the general guidelines for preventing direct identification as most Twitter users preferred.^26^ The focus needs to remain on the perceptions of LGBTQ+ health care which is achieved by replacing all usernames and in‐text references with hashtags. Another good practice is not connecting the tweet to a specific profile.^26^ The Twitter user could (possibly) be found when the tweet is searched for online, but this would only lead to the public profile of the Twitter users. The online and offline persona do not need to be the same.^18^, ^19^, ^27^

## 3 RESULTS

This exploratory supply‐based study was organized into the following categories that displayed perceptions of LGBTQ+ (centred) health care in the Twitter sample.

### 3.1 LGBTQ+ health beyond healthcare

The initial read‐through of the tweets showed an overrepresentation of political tweets, spearheaded by tweets on the Equality Act introduced by President Joe Biden. The tweets present the details of the Act as exemplified here:

@###### This is all the Equality Act does: it clarifies in law that LGBTQ people shouldn't be discriminated against on the basis of their gender or sexual orientation in employment, housing, public accommodations, education, or healthcare.

Subsequently, Arkansas (and other US states) introduced their bills to reduce the treatment for gender dysphoria for minors in the so‐called ‘Arkansas Save Adolescents From Experimentation Act’. The treatment for gender dysphoria in minors will not be covered by public health insurance which was one of the important details in the Equality Act from the President. In this case, Twitter users voiced their shock at this Act but also included misinterpretations of the content. A few tweets then responded to how these claims were overstretched as exemplified in this tweet:

@##### Actually, yes. (not endorsing the bill but providing context). The bill limits healthcare related to transitioning for transgender youth. Not general health care.

Even though the timing was chosen to increase the number of tweets on LGBTQ+ health care, the study focused on health care and aimed to understand it without the inclusion of the Act in Arkansas.

#### 3.1.1 Tweets in the Spanish language

The Spanish tweets (167) are from different countries and turned out to be an interesting (yet not completely useful) addition to the data as their focus on ‘gender' is mostly directed at women:

Women and girls of the world against: economic injustice, job uncertainty, unpaid care work, poverty, lack of health, lack of access to a livelihood and education, and an increase in violence based on gender.

Some tweets did focus on LGBTQ+ specifically such as this tweet from Mexico:

Today is Trans Awareness Day. Our country still does not guarantee that they have the same rights when it comes to marriage, starting a family, receiving health care or employment rights. No more unnecessary consultation or discussion. Gender Identity Law now! (Website)

There is specific attention to trans awareness, but this is not widely ingrained in the public Twitter agenda. Equality for women and girls needs to be widely established first according to the Spanish tweets in this study.

### 3.2 Health care is a human right

When the tweets are not directed at current political moves and players, a broader perspective is taken on the healthcare of all people. These tweets informed the audience about the human rights that are violated when there is no equal access to healthcare for all:

@###### The whole concept of denying health care to trans and other LGBTQ + is disgusting. Health care is a human right and trans rights are human rights. I don't understand how anyone can think otherwise.

@###### It's racism and discrimination 2.0. Transgender kids need health care just like the rest of us. Don't judge.

Connected to this topic are the tweets that focus their attention on healthcare professionals and their Hippocratic oath:

If I was a Dr in Arkansas I would be looking to leave the state in protest over this bill, which in my eyes forces Drs to abandon their oath.

Healthcare professionals themselves also participate in advocating the basics of health care:

I don't know how many times I need to say this, but here it is again: we take an oath to care for PEOPLE. No matter their race, sex, religion, etc. The LGTBQ+ community ARE PEOPLE. This is wrong and harmful for their health.

### 3.3 Health care for LGBTQ+ individuals

Many of the tweets concern trans health in particular. The bills, such as the one in Arkansas, are also specific to providing treatment for gender dysmorphia. Taking this treatment out of the health insurance is a direct attack and lack of acknowledgement of the necessity of quality health care and as this tweets exemplified here:

This is cruel & unusual punishment! My son began testosterone at 15. Before his transition he was a self‐harming suicidal wreck. It took over a year of counselling for the both of us and multiple tests and doc visits to start. It is not entered into lightly

It is irresponsible, reckless, and evil to deny anyone access to health care, especially during a worldwide pandemic. This is literally an attempt to kill transgender people in a legal way and it's happening in my country. This needs to be struck down immediately.

#### 3.3.1 Mental health

Something that is touched upon in these and many other tweets is the mental health care necessity of LGBTQ+ individuals as the suicide rate among this group is up to four times higher than their peers.^28^

Specifically, access to the right care is mentioned:

@##### @###### mental health care is already so taboo here but for people from lgbtq + communities….yeah. it's really scary to not be able to access available resources because they might not be accepting for you and. I am still way more privileged then so many. Yeah

I fear for my daughter and her LGBT+ friends and their access to appropriate medical and mental health care that is responsive to their needs. Add to it she's mixed race and it's even more complex. 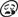

### 3.4 Person‐centred healthcare

The last section of the results goes into the secondary aim of uncovering how much of the tweets are focused on person‐centredness. Only four tweets (implicitly) mention PCC such as this example:

Good morning and Happy Doctors Day to everyone except for bigoted lawmakers who attempt to prevent us from providing equitable, patient‐centered care to #transgender youth, and physicians who refuse to do the same. You don't get to selectively apply the Hippocratic Oath.

PCC has been a buzzword in health care and is stated as the vision for good care on Twitter.^29^ However, it is only mentioned in less than 1% of the tweets in this sample.

## 4 DISCUSSION

This Twitter‐based study explored what people are tweeting about on the topic of LGBTQ+ (centred) health care. To capture a larger group of tweets, the topic was expanded to content containing mentions of LGBTQ+ and health care. The data collection was timed after the passing of the Equality Act in which discrimination against LGBTQ+ people was no longer permitted. Rather than a data set filled with tweets on how this Equality act would change access to health care, the set was overshadowed by a subsequent political debate. The results, therefore, leaned more towards a discussion on the legitimacy of prohibiting health care.

Twitter is often applied to discuss issues with a wider population.^14^ The tweets on LGBTQ+ health care were directed towards the rights of the individuals and the duties of healthcare professionals, organizations, and governments. This has also been found in tweets on gratitude during the Covid‐19 pandemic which showed how Twitter users directed their tweets to others on what ought to be the focus of press and public attention, notions that were proxies for ideological battles over roles and responsibilities.^30^

Another study on LGBTQ+ in Malaysia^31^ showed how the LGBTQ+ community is a topic for debate and most tweets contained a reference to being pro‐ or against the movement for more inclusivity. Although only a handful of tweets in this sample could be classified as against the Equality Act, the quest for more inclusivity was omnipresent. Much of the content is a referral to other people's work and a plea to get the audience educated on LGBTQ+ health care needs. Similar results were found when looking at PCC tweets in different languages where tweets focused on the vision of health care rather than the practical application.^29^

Central concepts in this study were performativity and imagined audience on Twitter.^13^, ^18^, ^19^ However, the performativity and self‐disclosure described by Kim et al.,^20^ which would be expected of this marginalized group, have remained at a minimum. This can be seen as a type of self‐preservation as the audience can be wildly inaccurate in their assessment of the Twitter user.^13^, ^32^, ^33^

Although there was a strong direction of perceptions, the actual message stayed neutral and mainstream such as mentioning human rights and the Hippocratic oath. In a study on social media posts of young people with low socioeconomic status, a similar strategy was observed where the participants only posted content that could make them look favourable.^33^ Tweeting about the necessity of LGBTQ+ health care makes the Twitter user look sympathetic without having to move from words to deeds.

Unlike the study on the #meToo movement,^21^ Twitter users did not divulge their experiences and most posts were about other people (known or unknown to the Twitter users). This might be as access to health care is a continuing struggle whereby disclosing personal perceptions can have adverse offline effects on access to quality care in the future.^1^, ^4^, ^5^

## 5 LIMITATIONS

The timing was chosen for the introduction of the Equality Act but turned out to not be ideal because the Arkansas bill and its discussion dominated the Twitter sample. This gave a false sense of there being many tweets that could help with the aim when it essentially showed the applicability of LGBTQ+ health care as a political statement.

There was no locational information in the data, so the tweets could be made from outside the United States as exemplified by the Spanish tweets. Nonetheless, as the Equality Act was only the starting point for this study, the perceptions of LGBTQ+ health care are presented regardless of the location.

The sample did not seem to include many Twitter users belonging to the LGBTQ+ community. Therefore, the experiences and perceptions of health care from this community stayed minimal. A different approach (like analyzing Twitter profiles) might have provided more of this data, although it would lose out on valuable perspectives from others.

The tweets are microblogs containing a maximum of 280 characters and by solely looking at the content that included the search terms, the context of the tweet is lost.

## 6 CONCLUSION

This study explored if the perception of Person‐Centred Care was mentioned concerning the LGBTQ+ community and health care on Twitter after the implementation of the Equality Act in the USA. The results showed that quality health care for LGBTQ+ individuals is still far from sufficient, and this challenge needs to be addressed first before person‐centredness can be found in the care. Optimistically, the increased access to quality care will coincide with increases in the implementation of more PCC in common practice to ensure that all people will receive care that is appropriate for their situation.

LGBTQ+ health‐related tweets are mostly used to express a standpoint in the political debate and argue their perception through general understandings such as human rights and the Hippocratic oath. The few examples that do disclose more personal content show how important access to quality health care and specifically PCC is for LGBTQ+ individuals.

With this study, the challenges came to light that LGBTQ+ health care is mentioned more to make a (political) standpoint than effectively addressing the inequality of care.

## CONFLICT OF INTEREST

The authors declare no conflict of interest.

## Supporting information

Supplementary information.

## Data Availability

The data that support the findings of this study are available on request from the corresponding author.

## References

[1] Bonvicini KA . LGBT healthcare disparities: what progress have we made. Patient Educ Couns. 2017;100(12):2357‐2361. 10.1016/j.pec.2017.06.003 28623053

[2] Stinchcombe A , Smallbone J , Wilson K , Kortes‐Miller K . Healthcare and end‐of‐life needs of lesbian, gay, bisexual, and transgender (LGBT) older adults: a scoping review. Geriatrics. 2017;2(1):13. 10.3390/geriatrics2010013 31011023 PMC6371094

[3] Rossi AL , Lopez EJ . Contextualizing competence: language and LGBT‐based competency in health care. J Homosex. 2017;64(10):1330‐1349. 10.1080/00918369.2017.1321361 28467155

[4] Scandurra C , Mezza F , Maldonato NM , et al. Health of non‐binary and genderqueer people: a systematic review. Front Psychol. 2019;10(June):1453. 10.3389/fpsyg.2019.01453 31293486 PMC6603217

[5] Aylagas‐Crespillo M , García‐Barbero Ó , Rodríguez‐Martín B . Barriers in the social and healthcare assistance for transgender persons: a systematic review of qualitative studies. Enferm Clín (Engl Ed). 2018;28(4):247‐259. 10.1016/j.enfcle.2017.09.005 29102529

[6] Ekman I , Busse R , van Ginneken E , et al. Health‐care improvements in a financially constrained environment. Lancet. 2016;387(10019):646‐647. 10.1016/S0140-6736(16)00285-3 26876711

[7] Lemoire SJ , Chen CP . Applying person‐centered counseling to sexual minority adolescents. J Couns Dev. 2005;83(2):146‐154. 10.1002/j.1556-6678.2005.tb00591.x

[8] Knutson D , Koch JM . Person‐Centered therapy as applied to work with transgender and gender diverse clients. J Humanist Psychol. 2022;62(1):104‐122. 10.1177/0022167818791082

[9] Håkansson Eklund J , Holmström IK , Kumlin T , et al. “Same same or different?” A review of reviews of person‐centered and patient‐centered care. Patient Educ Couns. 2019;102(1):3‐11. 10.1016/j.pec.2018.08.029 30201221

[10] Leplege A , Gzil F , Cammelli M , Lefeve C , Pachoud B , Ville I . Person‐centredness: conceptual and historical perspectives. Disabil Rehabil. 2007;29(20‐21):1555‐1565. 10.1080/09638280701618661 17922326

[11] Mead N , Bower P . Patient‐centredness: a conceptual framework and review of the empirical literature. Soc Sci Med. 2000;51(7):1087‐1110. 10.1016/S0277-9536(00)00098-8 11005395

[12] Johnson PR , Yang SU . Uses and Gratifications of Twitter. The Communication Technology Division of the Annual Convention of the Association for Education in Journalism and Mass Communication; 2009:1‐32.

[13] Marwick AE , Boyd D . I tweet honestly, I tweet passionately: Twitter users, context collapse, and the imagined audience. New Media Soc. 2010;13(1):114‐133. 10.1177/1461444810365313

[14] Krueger EA , Young SD . Twitter: a novel tool for studying the health and social needs of transgender communities. JMIR Ment Health. 2015;2(2):1‐8. 10.2196/mental.4113 PMC4465794 26082941

[15] Fox J , Ralston R . Queer identity online: informal learning and teaching experiences of LGBTQ individuals on social media. Comput Human Behav. 2016;65:635‐642. 10.1016/j.chb.2016.06.009

[16] Sinnenberg L , Buttenheim AM , Padrez K , Mancheno C , Ungar L , Merchant RM . Twitter as a tool for health research: a systematic review. Am J Public Health. 2017;107(1):e1‐e8. 10.2105/AJPH.2016.303512 PMC5308155 27854532

[17] Eysenbach G . Infodemiology and infoveillance: framework for an emerging set of public health informatics methods to analyze search, communication and publication behavior on the Internet. J Med Internet Res. 2009;11(1):e11. 10.2196/jmir.1157 19329408 PMC2762766

[18] Cover R . Performing and undoing identity online: social networking, identity theories and the incompatibility of online profiles and friendship regimes. Convergence. 2012;18(2):177‐193. 10.1177/1354856511433684

[19] Papacharissi Z . Without you, I'm nothing: performances of the self on Twitter. Int J Commun. 2012;6:1989‐2006. 10.1932/8036/20120005

[20] Kim J , Kim J , Collins C . First impressions in 280 characters or less: sharing life on Twitter and the mediating role of social presence. Telemat Inform. 2021;61(February 2020):101596. 10.1016/j.tele.2021.101596

[21] Bogen KW , Bleiweiss KK , Leach NR , Orchowski LM . #MeToo: disclosure and response to sexual victimization on Twitter. J Interpers Violence. 2021;36(17‐18):8257‐8288. 10.1177/0886260519851211 31117851

[22] Onanuga P . Coming out and reaching out: linguistic advocacy on queer Nigerian Twitter. J African Cult Stud. 2021;33(4):489‐504. 10.1080/13696815.2020.1806799

[23] Statistical Cybermetrics Research Group . Mozdeh [software]. 2014. Retrieved from 10.2196/mozdeh.wlv.ac.uk

[24] Graneheim UH , Lundman B . Qualitative content analysis in nursing research: concepts, procedures and measures to achieve trustworthiness. Nurse Educ Today. 2004;24(2):105‐112. 10.1016/j.nedt.2003.10.001 14769454

[25] Williams ML , Burnap P , Sloan L . Towards an ethical framework for publishing Twitter data in social research: taking into account users' views, online context and algorithmic estimation. Sociology. 2017;51(6):1149‐1168. 10.1177/0038038517708140 29276313 PMC5718335

[26] Fiesler C , Proferes N . “Participant” perceptions of Twitter research ethics. Soc Media Soc. 2018;4(1):1‐14. 10.1177/2056305118763366

[27] Subrahmanyam K , Reich SM , Waechter N , Espinoza G . Online and offline social networks: use of social networking sites by emerging adults. J Appl Dev Psychol. 2008;29(6):420‐433. 10.1016/j.appdev.2008.07.003

[28] Johns MM , Lowry R , Haderxhanaj LT , et al. Trends in violence victimization and suicide risk by sexual identity among high school students ‐ youth risk behavior survey, United States, 2015‐2019. MMWR Suppl. 2020;69(1):19‐27. 10.15585/mmwr.su6901a3 32817596 PMC7440203

[29] van Diepen C , Wolf A . “Care is not care if it isn't person‐centred”: a content analysis of how person‐centred care is expressed on Twitter. Health Expect. 2021;24(2):548‐555. 10.1111/hex.13199 33506570 PMC8077091

[30] Day G , Robert G , Leedham‐Green K , Rafferty AM . An outbreak of appreciation: a discursive analysis of tweets of gratitude expressed to the National Health Service at the outset of the COVID‐19 pandemic. Health Expect. 2021;25(August):149‐162. 10.1111/hex.13359 34543519 PMC8652934

[31] Naim M , Ali M , Mothar NM . Discourses on Twitter contribute to the concept of resilience in the Lgbt Community in Malaysia. ESTEEM J Soc Sci Human. 2020;5(February):27‐47.

[32] Marwick AE , Fontaine C , Boyd D . “Nobody sees it, nobody gets mad”: social media, privacy, and personal responsibility among low‐SES youth. Soc Media Soc. 2017;3(2). 10.1177/2056305117710455

[33] Pitcan M , Marwick AE , Boyd D . Performing a vanilla self: respectability politics, social class, and the digital world. J Comput Mediat Commun. 2018;23(3):163‐179. 10.1093/jcmc/zmy008

