# Supplementary material for: A content analysis on the perceptions of LGBTQ+ (centred) health care on Twitter
Source: Health Expect. 2022 Oct 17;25(6):3238–45. doi: 10.1111/hex.13631 (PMC9700160; doi:10.1111/hex.13631)
Supplement: Supplementary file 1 — Supplementary information. [file HEX-25--s001.docx]

| lgbtq centred care | gender minority centred care | transgender centred care |
| --- | --- | --- |
| lgbtq centered care | gender minority centered care | transgender centered care |
| lgtbq centred care | sexual minority centred care | transsexual centred care |
| lgtbq centered care | sexual minority centered care | transsexual centered care |
| lgbt centred care | gender minorities centred care | transgender health care |
| lgbt centered care | gender minorities centered care | transgender health care |
| lgbt centred caring | sexual minorities centred care | transsexual health care |
| lgbt centered caring | sexual minorities centered care | transsexual health care |
| lgbtq health care | gender minority health care |  |
| lgbtq health care | gender minority health care |  |
| lgtbq health care | sexual minority health care |  |
| lgtbq health care | sexual minority health care |  |
| lgbt health care | gender minorities health care |  |
| lgbt health care | gender minorities health care |  |
| lgbtq health caring | sexual minorities health care |  |
| lgbtq health caring | sexual minorities health care |  |
|  |  |  |
| atencion centrado lgbtq | tencion centrada minoria de genero | cuidado salud lgbtq |
| atencion centrado lgbt | atencion centrada lgbtq | cuidado salud lgbt |
| atencion centrado transexual | atencion centrada transexual | cuidado salud transexual |
| atencion centrada minoria sexual | atencion centrado transgenero | cuidado salud minoria sexual |
| cuidado centrado lgbtq | atención médica género | cuidado salud transgenero |
| cuidado centrado lgbt | cuidado de la salud género | cuidado salud minoria de genero |
| cuidado centrado transexual | atencion salud lgbtq | atencion salud minoria de genero |
| cuidado centrado minoria sexual | atencion salud lgbt | atencion salud lgbtq |
| cuidado centrado transgenero | atencion salud transexual | atencion salud transexual |
| cuidado centrado minoria de genero | atencion salud minoria sexual | atencion salud transgenero |

**Appendix 1. English and Spanish search terms for Application Programming Interface**
